# Improvement of PD-1 Blockade Efficacy and Elimination of Immune-Related Gastrointestinal Adverse Effect by mTOR Inhibitor

**DOI:** 10.3389/fimmu.2021.793831

**Published:** 2021-12-20

**Authors:** Xin Bai, Xueyan Wang, Guozhen Ma, Jinen Song, Xiaowei Liu, Xi Wu, Yujie Zhao, Xu Liu, Zhihui Liu, Wei Zhang, Xin Zhao, Zirui Zheng, Jing Jing, Hubing Shi

**Affiliations:** ^1^ Laboratory of Integrative Medicine, Clinical Research Center for Breast, State Key Laboratory of Biotherapy, West China Hospital, Sichuan University and Collaborative Innovation Center, Chengdu, China; ^2^ Microbial Innovation and Development Department, Chemical Manufacturing and Control (CMC) Center, Hangzhou Zhong Mei Hua Dong Pharmaceutical Co., Ltd., Hangzhou, China

**Keywords:** immune checkpoint blockade, immune-related adverse events, mTOR inhibitor, effector T-cells, melanoma

## Abstract

During the past decades, immunotherapy, especially the antibody-mediated immune checkpoint blockade (ICB) has shown durable tumor inhibition and changed the paradigm of cancer treatment. However, a growing body of evidence suggests that ICB treatment induces severe immune-related adverse events (irAEs), and the side effect even leads to the discontinuation of lifesaving treatment. Here, we found that ICB treatment induces colitis in melanoma patients and promotes the infiltration of CD8^+^ effector T cells into colitic lesions. Further transcriptomic dissection indicated the PI3K-AKT-mTOR pathway was highly activated in CD8^+^ effector T cells of colitic lesions. Moreover, we developed a mouse melanoma model to recapitulate the gastrointestinal toxicity of anti-PD-1 treatment in clinical settings. Anti-PD-1 treatment significantly contributed to the infiltration of CD8^+^ T cells, and correspondingly induced severe enteritis. Immunohistochemistry experiments showed that the PI3K-AKT-mTOR pathway of T cells was activated by anti-PD-1 treatment. Blockade of the pathway with mTOR inhibitor sirolimus not only inhibits tumor growth but also suppresses the T cell infiltration in colitic lesions. More importantly, combination with sirolimus and anti-PD-1 synergistically inhibits tumor growth *via* inducing the immunogenic cell death of tumor cells *in vivo*. In summary, our research demonstrated the principle of mTOR inhibitor and anti-PD-1 combinatorial therapeutic regimen, which provided a novel therapeutic strategy for irAEs in clinics.

## Introduction

Cancer immunotherapies are broadly defined as therapies that directly or indirectly target any component of the immune system involved in the anti-cancer immune response (1). Immune-checkpoint blockade is one class of these therapies that is able to achieve durable responses on multiple types of cancer (2). Normally, immune checkpoints such as PD-1 and CTLA-4 pathways downregulate T cell responses to control the immune system and thus protect the body from possibly damaging responses, tumor cells can use this system to avoid immune cells’ attack through the activation of immune checkpoints and inhibition of the T cell responses (3, 4). Agents such as nivolumab/pembrolizumab targeting programmed cell death protein 1 (PD-1) or ipilimumab targeting cytotoxic T-lymphocyte -associated protein 4 (CTLA-4) activate T cells by inhibiting the interaction of T cell surface proteins (PD-1/CTLA-4) with their respective ligands on antigen presenting cells and tumor cells (5, 6). However, these activations of the immune system can lead to inflammatory side effects, which are unique and are different from those of traditional cancer therapies, and have a delayed onset and prolonged duration (7). These side effects are often termed as immune-related adverse events (irAEs), which can involve any organ or system and often share similarities with autoimmune inflammatory diseases (8, 9).

Increased use of immune checkpoint inhibitors (ICI) has resulted in increased reports of irAEs, which have been reported to occur in up to 80% of patients receiving ICI therapy. Among these patients, irAEs most typically impact the skin, GI tract, and endocrine, but they can also affect the lung and cardiovascular system which were rare but more severe and lethal. Several guidelines on the management of irAEs have been published (10, 11). Despite the great and often durable clinical benefits of the immune checkpoint blockade therapy, patients have to hold immune checkpoint blockade therapy if severe irAEs occur, high-dose and systemic corticosteroids were administered when the toxicity does not resolve. Corticosteroids were known to induce apoptosis of proliferating T cells and may potentially diminish optimal therapeutic responses (12, 13). Thus, the optimum treatment option for irAEs is still a matter of debate. To find alternative treatment approaches that not only inhibit irAEs but also enhance anti-tumor immunity, at least not antagonistic, is therefore of crucial importance.

The precise mechanisms underlying irAEs are currently unknown, however, given the pivotal role of immune checkpoints in maintaining immunologic homeostasis, it is believed that during systemic administrations of ICIs, effector T cells in the tumor microenvironment were activated to exert anti-tumor responses, while those effector T cells out of tumor microenvironment were also over-activated and may exert toxic effects on healthy tissues (14). T cells activated by ICIs and reactive to tumor cells may also be inflamed and kill normal cells in healthy tissues, as evidenced by the shared TCR repertoire between tumors and other irAEs affected normal tissues (15, 16).

In this study, we re-analyzed the single-cell sequence data of colon biopsies from ICIs-treated patients who underwent colitis, the bioinformatic results indicated that infiltration and activation of PI3K-AKT-mTOR pathway in colon CD8^+^ T cells may play a critical role in patients with irAEs. Then, to recapitulate the gastrointestinal toxic effects of anti-PD-1 treatment in humans, we established a pre-clinical mouse model. Anti-PD-1 treatment induced severe enteritis by promoting CD8^+^ T cells infiltration and activating PI3K-AKT-mTOR pathway in T cells. We found that using an mTOR inhibitor dramatically reduces the gastrointestinal toxicity caused by anti-PD-1 treatment. Importantly, we found that mTOR inhibitor not only reduces enteritis but also synergistically improved the anti-tumor immune response by triggering the immunogenic cell death (ICD) of tumor cells. Taken together, our findings provide a new strategy that avoids ICI induced irAE and synergizes immunotherapy.

## Materials and Methods

### Pre-Process of scRNA-Seq Data

Single cell RNA sequence data of this study were downloaded from the GEO database (GSE144469). Raw data were aligned to GRCh38 genome reference using 10X Genomics Cell Ranger (v3.0.2). Then, we removed cells with high expression levels of mitochondrial genes, for CD3^+^ dataset: 11.13%; for CD45^+^ dataset: 13.33%. Second, cells that expressed fewer than 200 genes were detected. Third, potential doublets were identified and removed by using DoubletFinder (v2.0.2), using 92.5th percentile of the doublet score as the cutoff. Finally, a total of 68,043 single cells remained for the CD3^+^ datasets.

### Annotation of Cell Clusters and Data Visualization

We performed unsupervised clustering and differential gene expression analyses in the Seurat (v4.0.1, R package). Genes expressed in less than 10 cells were filtered, and the UMI count matrix was normalized by using ‘NormalizeData’ method. Based on the normalized gene expression matrix, highly variable genes were identified by using the ‘FindVariableFeatures’ function with the ‘vst’ method. For the clustering analysis of the CD3^+^ dataset, TCR genes (TRAV, TRBV, TRDV, and TRGV genes) were removed to avoid the dominant effect, and the first 20 principal components were Selected. To analyze CD3^+^ T cells in detail, CD3^+^ T cells were split into CD4^+^ and CD8^+^ T cell subsets according to the mutually exclusive expression of CD4 and CD8A genes (CD4^+^: CD4 > 1 and CD8A < 1; CD8^+^: CD4 < 1 and CD8A > 1). Then, CD8^+^ cells were clustered as the pipelines described above, and cell clusters were annotated with the well-studied marker genes of different immune cell types.

### Functional Enrichment Analysis

SingleCellSignatureExplorer v3.6 was used to compute single-cell signature scores of P1 and P2 epithelial cells. This software computes in each cell a score for any gene set. C2.CP.KEGG, C5. BP, and C6 gene sets of MSigDB v7.1 were used here. The score of gene set GSx in the cell Cj was computed as the sum of all UMI for all the GSx genes expressed in Cj, divided by the sum of all UMI expressed by Cj. As gene numbers in each gene set are highly variable, a single cell score for a signature cannot be compared to that of other signatures.

### Pseudotime Analysis

The single-cell trajectories were constructed by Monocle3 (V0.2.3.0, R package). Monocle learns the sequence of gene expression changes each cell must go through as part of a dynamic biological process. Then it constructs a trajectory that mainly reflects the progress of cells moving from the starting state. Firstly, we created a CellDataSet object for CD8^+^ single-cells of each cell type. Next, UMAP was used to reduce the dimensionality of the CellDataSet with reduce_dimension function. Then we cluster cells by cluster_cells function, each cell is assigned not only to a cluster but also to a partition, which is helpful to trajectories learning. To learn the trajectory graph, the learn_graph function was used to fit a principal graph within each partition: After we’ve learned a graph, the order_cells function was used to calculate where each cell falls in pseudotime. We set cluster 6_CM_NaÏve as the root node of the trajectory graph by get_earliest_principal_node function.

### Cells and Mice

SMM103 cell line was derived from transgenic BRAF^V600E/wt^, CDKN2^-/-^, PTEN^-/-^ mouse as previously described (17, 18). All cells were cultured with complete DMEM medium containing 10% FBS, 100U/ml penicillin and 100ug/ml streptomycin. All cells were cultured at 37 °C in a humidified, 5% CO_2_ incubator. Cells were routinely tested for mycoplasma contamination using one-step Quickcolor Mycoplasma detection Kit (Shanghai Tise Medical Technology Co., Ltd., China, # MD001). Female C57BL/6 mice (specific pathogen-free conditions) were purchased from Beijing Vital River Laboratory Animal Technology Co., Ltd. (Beijing, China). The animals were housed and maintained under specific pathogen-free conditions in facilities and treated humanely throughout the studies. All animal experiments were performed according to protocols approved by the Ethics Review Committee of Animal Experimentation of Sichuan University. All the animal-handling procedures were performed according to the Guide for the Care and Use of Laboratory Animals of the National Institutes of Health and followed the guidelines of the Animal Welfare Act. Mouse melanoma model was generated in 8-week-old C57BL/6 mice by subcutaneous implantation of 1×10^6^ cells. Mice were randomized into groups for collection and analysis. Tumor volume was calculated by the formula (tumor volume = 0.52 × length × width^2^). When tumors reached a volume of approximately 80 mm^3^, mice were treated with saline, anti-PD-1 (5mg/kg, *i.v.*), sirolimus (2mg/kg, *i.p.*).

### Cell Proliferation Assay

The MTT (3-[4,5-dimethylthiazole-2-yl]-2,5-diphenyltetrazolium bromide) assay was used to examine cell viability. SMM103 cells (1500/well) were seeded into 96-well plate with 50ul of medium and maintained in an incubator at 37 °C in a humidified, 5% CO2 atmosphere. After culturing for 24 h, cells were treated with 50ul of culture medium containing various concentrations of sirolimus (a kindly gift from Hangzhou Zhong Mei Hua Dong Pharmaceutical CO., LTD) (0, 0.01, 0.1, 1, 10, 100 nM) or rapamycin (Selleck, S1039) (0, 0.01, 0.1, 1, 10, 100 nM) for 72 h or treated with 50ul of culture medium captaining 100nM sirolimus or rapamycin at various timepoints (0, 3, 6, 24, 48, 72 h). Five multiple wells were set for each sample. Subsequently, 15ul of dye solution was added to each well and incubated for 4 h. The 96-well plate was read at 570nm absorbance by a microplate reader.

### Western Blot

SMM103 cells (1000/well) were seeded into 6-well plate, after culturing for 24 h, cells were treated with various concentrations of sirolimus (0, 0.01, 0.1, 1, 10, 100 nM) for 72 h or treated with 100 nM sirolimus at various timepoints (0, 3, 6, 24, 48, 72 h). Afterward, the cells were washed with cold PBS and lysed in cold RIPA buffer (20-188, Merck Millipore) supplemented with protease inhibitor (Bimake, B14001) and phosphatase inhibitor (Bimake, B15002). Following antibodies were used: Calreticulin antibody (abcam, 1:1000, ab92516), HSP70 antibody (abcam, 1:1000, ab5439), phosphor-AKT (Ser 473) antibody (CST, 1:100, 4060L), phosphor-S6 (Ser240/244) antibody (CST, 1:1000, 5364s), beta-actin antibody (Zsbio, 1:2000, TA-09).

### Immunohistochemistry and Immunofluorescence

All the tissues from mouse were fixed in 10% formalin and processed for paraffin embedding. FFPE samples were sectioned (4 μm) and mounted on microscope slides (Mevid, PC4-301-16). Slides were baked at 60°C overnight and deparaffinized in 3 changes of xylene, and then rehydrated in 2 changes of 100% ethanol followed by a series of 95, 95 and 85% ethanol and distilled water. Antigen retrieval was performed by heating slides to 95°C for 15min in a microwave followed by a 1 h cool down at room temperature. Slides were then washed with PBS, endogenous peroxidases were inactivated by 3% hydrogen peroxide for 10 min. Slides were washed with PBS and then incubated with primary antibodies at 4°C overnight. For immunohistochemistry, slides were washed with PBS and then incubated with HRP-conjugated secondary antibodies at room temperature for 45 min, after being washed with PBS, the slides were incubated with DAB for visualization. For immunofluorescence, slides were washed with PBS and then incubated with fluorescence-conjugated secondary antibodies at room temperature for 1 h (protect from light), after washed with PBS, the slides were stained with DAPI nuclear dye. Cell densities (number of positive cells mm^-2^) were calculated using image J software. Primary antibodies: CD3 (abcam, 1:100, ab11089), CD8 (abcam, 1:2000, ab217344), GZMB (abcam, 1:3000, ab255598), CD31 (CST, 1:100, 77699), phosphor-AKT (Ser 473) antibody (CST, 1:100, 4060L), phosphor-S6 (Ser240/244) antibody (CST, 1:1000, 5364s), Calreticulin antibody (abcam, 1:250, ab92516), HSP70 antibody (abcam, 1:2000, ab5439). For detection of apoptosis, the slides were stained with anti-CD3 antibody and stained with DeadEnd Fluorometric Tunel Kit following the manufacturer’s protocol (Promega, TB235).

## Results

### T Cells Activation and Infiltration Results in ICI Treatment Induced Colitis

**Figure 1:**
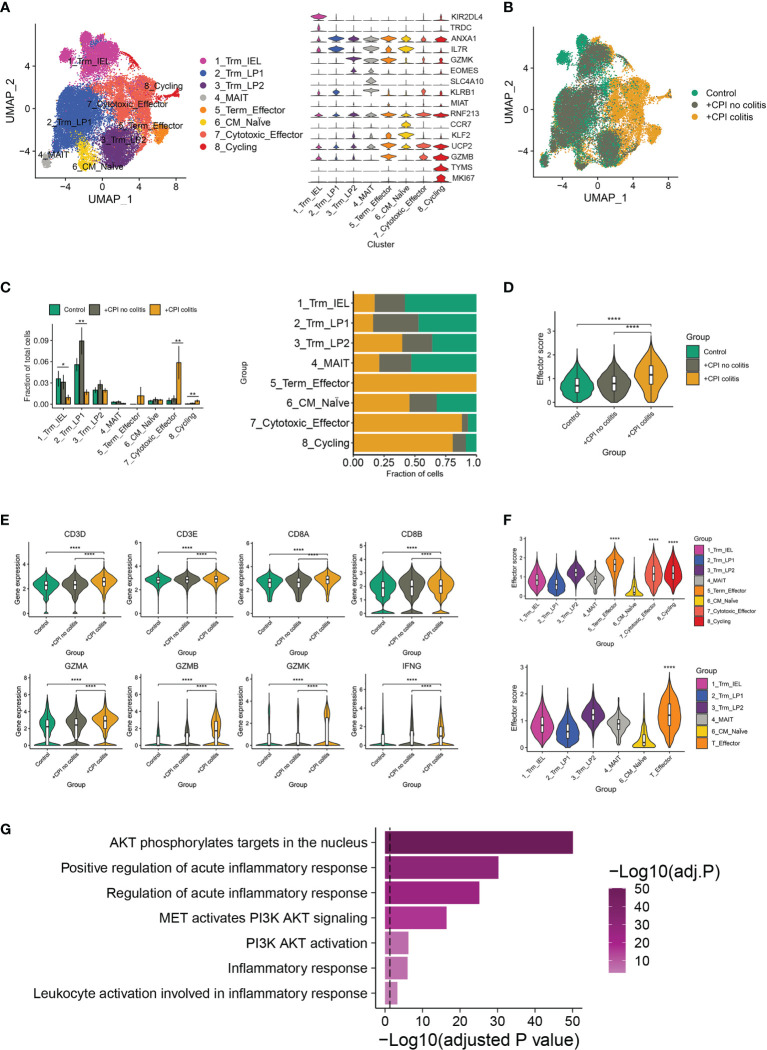
irAEs-related changes in T cell sub-populations and mTOR pathways. **(A)** Identification of colon CD8^+^ T cell clusters across all samples (left). Violin plots (right) showing the expression levels of cluster-specific marker genes in CD8+ T cells. Rows represent genes, and columns represent clusters. Each violin indicates the gene expression distribution within this cluster. **(B)** Distribution of CD8^+^ T cells across clusters among patient groups. (n=6-8 patients per group). **(C)** Quantification of cell cluster frequency representation (left) and distribution of cell clusters (right) among patient groups. *p < 0.05; **p < 0.01, analyzed by two-sided Wilcoxon test comparing control and colitis groups, average and SEM are shown for each patient group. **(D)** Effector score of CD8^+^ T cells among patient groups. ****p < 0.0001, two-sided Wilcoxon test. **(E)** Violin plots showing the expression of selected effector marker genes among patient groups. ****p < 0.0001, two-sided Wilcoxon test. **(F)** Effector scores computed for each CD8^+^ T cell sub-cluster (top) and effector T cell cluster (down). ****p < 0.0001, two-sided Wilcoxon test. **(G)** Gene set enrichment analysis of effector T cells and other CD8^+^ T cells. Method used to adjust the p-values is B-H.

As a previous study, an accumulation of CD8^+^ T cells was observed in ICI-induced colitis patients (15). To further identify the major changes in CD8^+^ T cells composition, effector programs, and molecular pathways underlying colitis induced by ICI treatment, we re-analyzed the single-cell RNA sequence data from healthy donors, ICI-treated melanoma patients with colitis or without colitis (GEO: GSE144469) (15). Eight well-defined sub-populations of CD8^+^ T cells were annotated based on published signatures and cell markers (**Figure 1A**). We found significant differences in the CD8^+^ T cell sub-populations between patients with or without colitis who were treated with ICI and healthy donors (**Figure 1B**). Three clusters, including cluster 5 (Terminal effector T cells), cluster 7 (cytotoxic effector T cells) and cluster 8 (cycling T cells), were almost exclusively enriched in ICI-treated colitis patients (**Figure 1C**). Notably, the effector score, and index of immune effector genes, were higher in CD8^+^ T cells from +CPI colitis patients compared with both control groups, suggesting a stronger cytotoxic function of CD8^+^ T cells in colitic lesions (**Figure 1D**). Cytotoxicity-related genes, including CD3D, CD3E, CD8A, CD8B, GZMA, GZMB, GZMK, and IFNG, were all upregulated in ICI-treated colitis patients (**Figure 1E**). These results suggest that the enrichment of three “effector clusters” (clusters 5,7 and 8) was the main reason that caused colitis in ICI-treated patients. Indeed, these effector clusters showed a high effector score compared with other clusters (**Figure 1F**). To identify the molecular mechanisms underlying colitis induced by these effector clusters, we performed gene set enrichment analysis, and PI3K-AKT-mTOR signatures showed upregulation in effector T cells (**Figure 1G**). Considering PI3K-AKT-mTOR pathway is crucial in T cell activation and function, we hypothesized that anti-PD-1 may activate the PI3K-AKT-mTOR pathway of T cells and promote the infiltration of T cells in colitic lesions.

### Anti-PD-1 Treatment Induced irAEs in Mouse Melanoma Model

**Figure 2:**
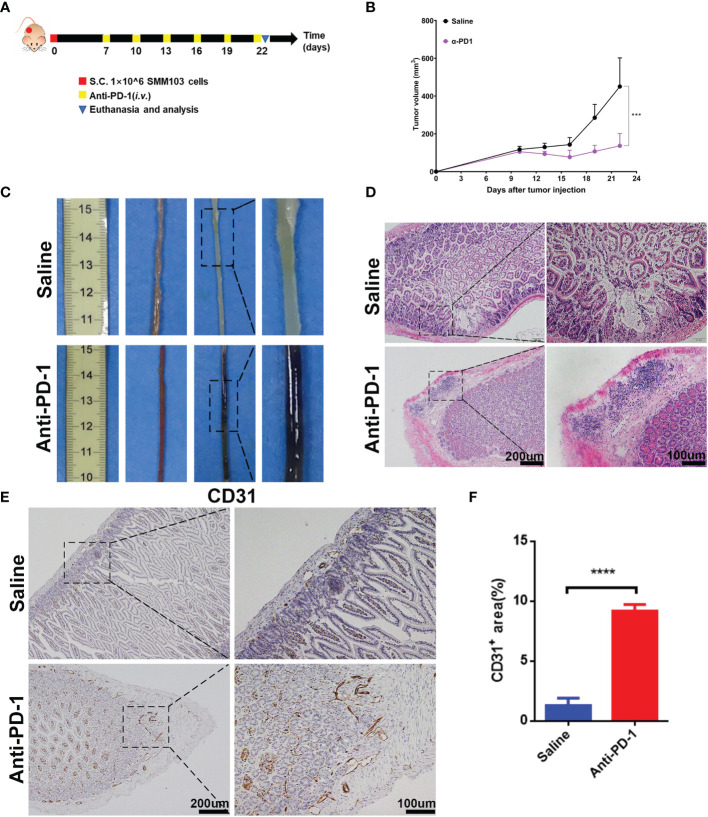
Establishment of irAEs mouse model. **(A)** Representative diagram showing experimental scheme of establishment of irAEs mouse model. Immune competent C57BL/6 mice were subcutaneously inoculated with 1×10^6^ SMM103 melanoma cells on day 0. When tumor volume achieved ≈80 mm^3^, mice were treated with saline or anti-PD-1, respectively. Details of treatment are provided in the Methods section. **(B)** The average tumor growth curves of mice treated with saline and anti-PD-1, respectively. The results are presented as mean ± SD (n=5), ***p < 0.001. **(C)** Representative intestine from each group after euthanizing the mice on day 22. **(D)** Lymphatic infiltration in intestine detected by staining with hematoxylin and eosin (H&E). Scale bar 200 μm **(E)** The formation of new blood vascular was visualized by CD31 IHC staining. Scale bar 200 μm. **(F)** Quantitative results of CD31 positive area. Data was expressed as mean ± SD (n=4), ****p < 0.0001.

To illuminate the correlation between irAEs and immunotherapy, we recapitulated the clinical therapeutic process in a mouse model. SMM103, an anti-PD-1 antibody responsive cell line, was subcutaneously inoculated in immune competent C57BL/6 mice, the mice were randomly divided into two groups when the tumor grew up to ≈80 mm^3^, and were treated with saline and anti-PD-1 antibody (5mg/kg), respectively (**Figure 2A**). Tumor volumes were recorded every 3 d, and tumor growth curves were plotted during treatment (**Figure 2B**). With the anti-PD-1 treatment, the tumor growth was significantly inhibited, indicating this model is suitable for studying the irAEs. After the treatment of anti-PD-1 for 9 days, mucus in the stool was observed in some mice, indicating that the intestinal canal became dysfunctional. In addition, pruritus and convulsion were also common phenomena during drug treatment. By day 18, the mice were of poor health status. Consequently, the mice were sacrificed, and the intestine were harvested for analysis. Severe hemorrhages were observed in the intestine from mice treated with anti-PD-1, but not in the control group (**Figure 2C**). Compared with the control group, Haematoxylin and eosin (H&E) analysis in intestine showed a significant increase in immune cells infiltrated into the intestine from anti-PD-1 group (**Figure 2D**). Immunochemistry analysis of CD31 in intestine demonstrated that the new blood vessels were formed (**Figure 2E**). To characterize the structural and morphological of blood vessels in intestine, the area of CD31^+^ staining was carefully measured at each mouse (**Figure 2F**). The area of CD31^+^ staining was significantly increased in intestine from anti-PD-1 treatment group, which is in line with the phenomena of gastrointestinal hemorrhage.

### Increase in CD8^+^ T Cells and Activation of mTOR Pathways During Anti-PD-1 Treatment

**Figure 3:**
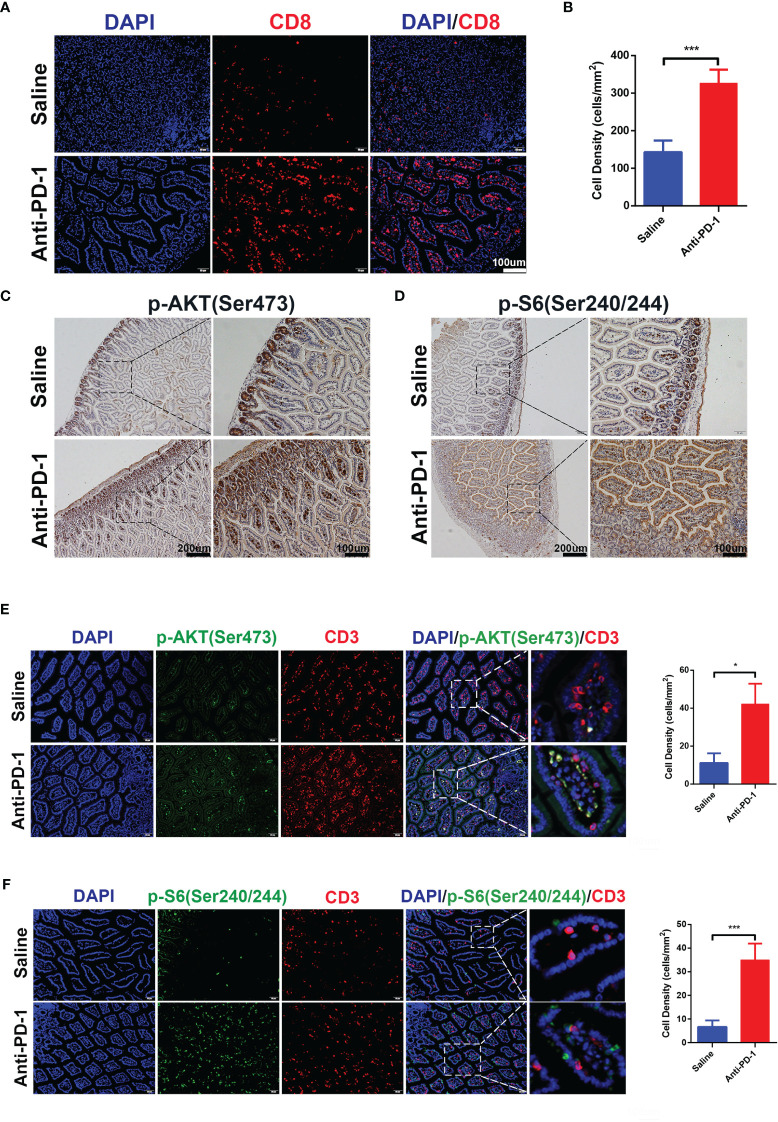
CD8^+^ T cells infiltration and activation of PI3K-AKT-mTOR pathway were induced by anti-PD-1 immunotherapy. **(A)** Immunofluorescence staining showed the infiltration of CD8^+^ T cells in colon. CD8 is shown in red and DAPI in blue. Scale bar 100 μm. **(B)** The densities of CD8^+^ T cells in each group were quantified, the results were presented as mean ± SD (n=4), ***p < 0.001. **(C)** Phosphorylation of AKT (Ser 473) was detected by IHC staining. Scale bar 200 μm. **(D)** Phosphorylation of S6 Ribosomal Protein (Ser 240/244) was detected by IHC staining. Scale bar 200 μm. **(E)** Double immunofluorescent staining with CD3 and phosphor-AKT (Ser 473) antibodies counterstained with DAPI on FFPE colon sections. CD3 is shown in red, phosphor-AKT (Ser 473) in green, and DAPI in blue. Scale bar 100 μm. **(F)** Double immunofluorescent staining with CD3 and phosphor-S6 Ribosomal Protein (Ser 240/244) antibodies counterstained with DAPI on FFPE colon sections. CD3 is shown in red, phosphor-S6 Ribosomal Protein (Ser 240/244) in green, and DAPI in blue. Scale bar 100 μm. *p < 0.05.

Initial bioinformatic analysis of scRNA-seq data showed that, compared with healthy donors, the striking increase of CD8^+^ effector T cells in the colon was obvious in checkpoint inhibition therapy treated melanoma patients with colitis. To investigate whether the increase of CD8^+^ T cells can be reproduced in our *in vivo* model, we detected the CD8^+^ T cells in the colon tissues by immunofluorescence (**Figures 3A, B** **)**. The results indicated the infiltration of CD8^+^ T cells in colon tissues was promoted by anti-PD-1, which is consistent with bioinformatic analysis and clinical observation. As above results, ICI treatment induced the hyperactivation of PI3K-AKT-mTOR pathway in CD8^+^ T cells (**Figure 1G**). To validate the activation of PI3K-AKT-mTOR pathway in colon tissues of anti-PD-1 treated mice, the phosphor-AKT (Ser 473) and phosphor-S6 (Ser 240/244) were characterized by IHC staining (**Figures 3C, D** **)**. The results showed that the phosphorylation of AKT and S6 were up-regulated by anti-PD-1treatment, indicating the upregulation of PI3K-AKT-mTOR pathway in colon tissues. Furtherly, we detected the phosphor-AKT and phosphor-S6 along with T cells marker CD3 by immunofluorescence (**Figures 3E, F** **)**. The phosphor-AKT and phosphor-S6 signals were observed in CD3^+^ T cells of colon tissues treated with anti-PD-1. These results indicate that we successfully developed the irAEs mouse model, and administrations of anti-PD-1 led to the recapitulation of the features associated with human irAEs. The results are also consistent with our bioinformatics analysis, enrichment of effector T cells in colon and upregulation of PI3K-AKT-mTOR pathway of these T cells may be the main reasons which caused colitis.

### Combination of Sirolimus and Anti-PD-1 Synergistically Inhibits Tumor Growth and Relieve irAEs *In Vivo*

**Figure 4:**
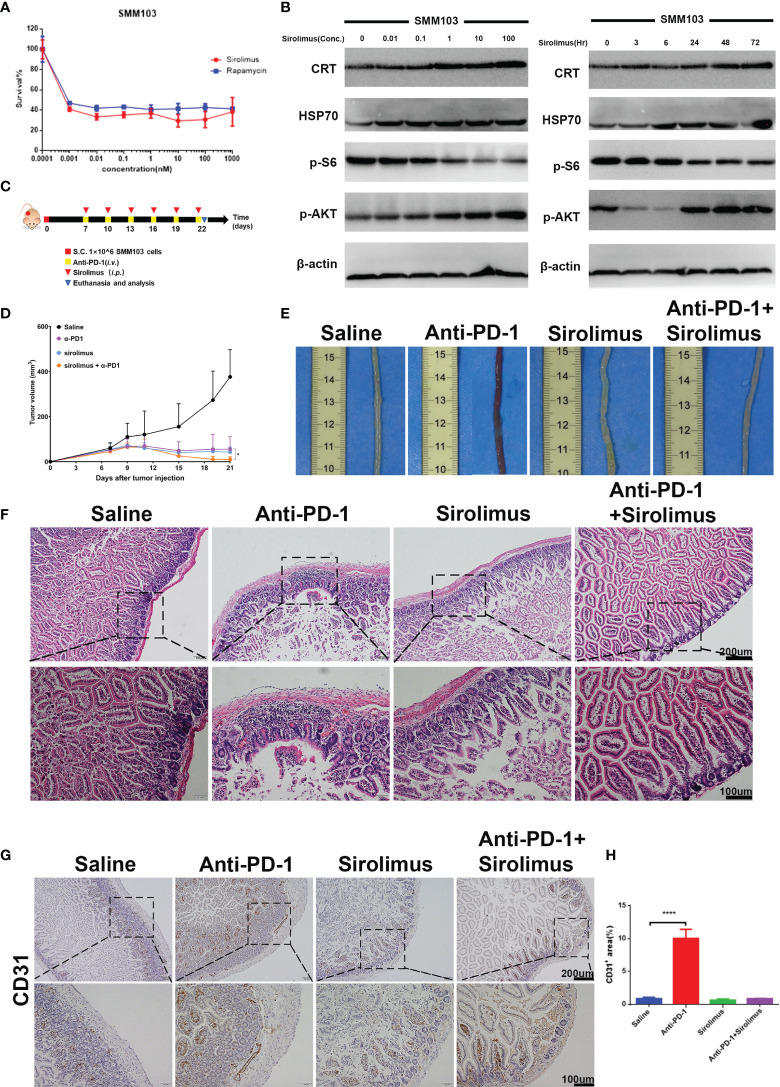
Sirolimus inhibited tumor cells growth and induced anti-inflammatory effects *in vivo*. **(A)** Three-day MTT survival assays of SMM103 melanoma cell line in response to sirolimus and rapamycin (as standard, all in nM). Results were expressed as mean ± SD (n=5). Data normalized to DMSO-treated controls. **(B)** Western Blot analysis of ICD-associated proteins, phosphor-AKT and phosphor-S6 Ribosomal Protein in SMM103 cell line in response to increasing concentrations of Sirolimus (72 h) (left). Western Blot analysis of ICD-associated proteins, phosphor-AKT, phosphor-S6 Ribosomal Protein in SMM103 cell line in response to sirolimus (100 nM) at indicated time points (3, 6, 24, 48, 72 h). **(C)** Immune competent C57BL/6 mice were subcutaneously inoculated with 1×10^6^ SMM103 melanoma cells on day 0. When tumor volume achieved ≈80 mm^3^, mice were treated with saline, anti-PD-1, sirolimus, anti-PD-1 plus sirolimus, respectively. Details of treatment are provided in the Methods section. **(D)** The average tumor growth curves of mice from each group. **(E)** Representative intestine from each group after euthanizing the mice on day 22. **(F)** H&E staining of FFPE colon sections from each group. Scale bar 200 μm. **(G)** CD31 IHC staining of FFPE colon sections from each group. Scale bar 200 μm. **(H)** Quantitative results of CD31 positive area. Data was expressed as mean ± SD (n=4), ****p < 0.0001.

Sirolimus is a specific mTOR inhibitor and it was reported to induce the durable cytostatic effects in melanoma. We tried to investigate whether sirolimus can eliminate colitis and maintain the anti-tumor effects at the same time. SMM103 melanoma cell toxicities of sirolimus and rapamycin (as standard, purchased from Selleck) were evaluated by MTT assay (**Figure 4A**), the results showed that sirolimus can suppress tumor cell growth. The phosphor-AKT and phosphor-S6 of the SMM103 melanoma cells treated with sirolimus were detected by western blot, SMM103 cells were exposed to various concentrations of sirolimus for 72 hours, sirolimus inhibited the phosphor-S6 at Ser240/244 and activated the phosphor-AKT at Ser473, starting at doses as low as 0.1nM (**Figure 4B**, left). We also treated SMM103 melanoma cells with 100 nM sirolimus at different time points, the inhibition of the phosphor-S6 was detected at 3 hours and last at least for 72 hours, phosphor-AKT was inhibited at first and then re-activated after 24 hours (**Figure 4B**, right). Immunogenic cell death (ICD) is a particular modality of cell death. Dying, stressed or injured cells can expose or release molecules on their surface or into the tumor microenvironment, which facilitates the activation and maturation of antigen-presenting cells and then priming of antigen-specific cytotoxic T lymphocytes (CTLs). This process was mainly mediated by damage-associated molecular patterns (DAMPs), which include calreticulin (CRT), heat shock protein 70 (HSP70), and other molecules. Results showed that CRT and HSP70 were upregulated after SMM103 melanoma cells were treated with sirolimus (**Figure 4B**), indicating that ICD was induced by sirolimus. Based on *in vitro* performance of sirolimus on PI3K-mTOR pathway inhibition and SMM103 tumor cells suppression, we then evaluated the efficacy of sirolimus *in vivo*. Immune competent C57BL/6 mice were subcutaneously inoculated with SMM103 melanoma cells, the mice were randomly divided into four groups when the tumor grew up to ≈80 mm^3^ and were treated with saline, anti-PD-1 (5mg/kg, *i.v.*), sirolimus (2mg/kg, *i.p.*), anti-PD-1 plus sirolimus, respectively, as depicted on experiment scheme (**Figure 4C**). Tumor growth curves were plotted during treatment (**Figure 4D**). The representative gross pictures of colon from all groups of mice have been shown (**Figure 4E**), severe hemorrhages were common phenomena in mice treated with anti-PD-1 monotherapy, and it was not seen in the control, sirolimus monotherapy, and combination group. H&E showed immune cells infiltration was evident in the colon of mice receiving anti-PD-1 monotherapy, the addition of sirolimus tended to relieve the infiltration (**Figure 4F**). Consistent with these findings, immunohistochemical detection of CD31 in colon tissues showed that anti-PD-1-induced increases of vessel destruction were obvious in mice that received anti-PD-1 monotherapy, but these effects were reduced by sirolimus treatment (**Figures 4G, H** **)**.

### Sirolimus Combined With Anti-PD-1 Decrease Infiltration and mTOR Pathways Activation of T Cells

**Figure 5:**
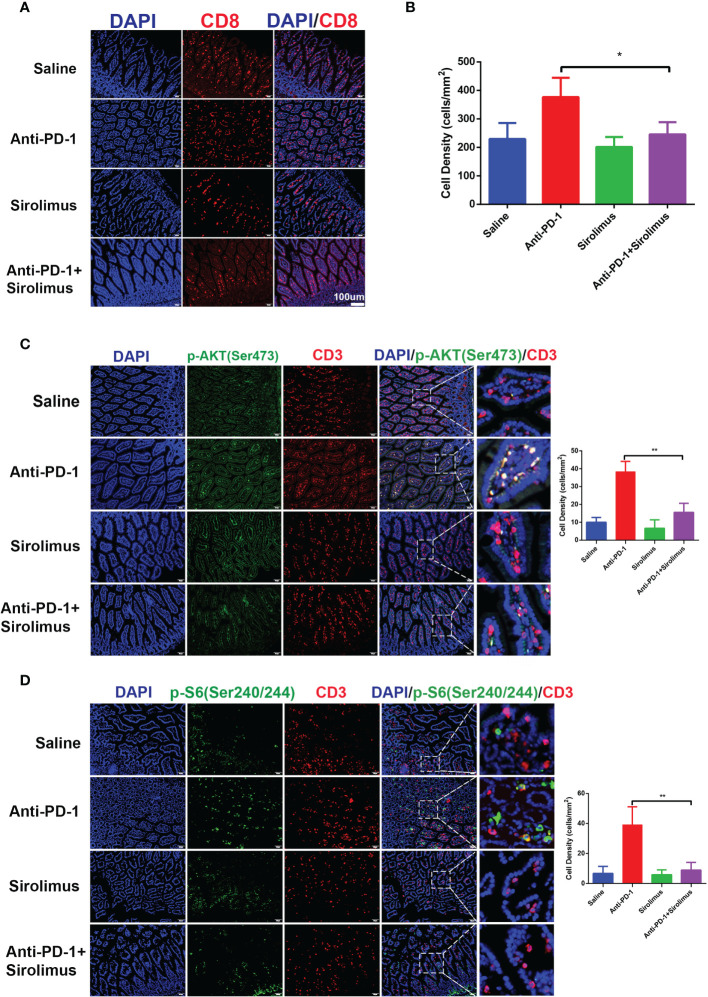
CD8^+^ T cells infiltration and activation of PI3K-AKT-mTOR pathway were reduced by sirolimus. **(A)** Immunofluorescence staining showed the infiltration of CD8^+^ T cells in colon was reduced by sirolimus. CD8 is shown in red and DAPI in blue. Scale bar 100 μm. **(B)** The densities of CD8^+^ T cells in each group were quantified. The results were presented as mean ± SD (n=4). *p < 0.05. **(C)** Double immunofluorescent staining with CD3 and phosphor-AKT (Ser 473) antibodies counterstained with DAPI on FFPE colon sections. CD3 is shown in red, phosphor-AKT (Ser 473) in green, and DAPI in blue. Scale bar 100 μm. **p < 0.01. **(D)** Double immunofluorescent staining with CD3 and phosphor-S6 Ribosomal Protein (Ser 240/244) antibodies counterstained with DAPI on FFPE colon sections. CD3 is shown in red, phosphor-S6 Ribosomal Protein (Ser 240/244) in green, and DAPI in blue. Scale bar 100 μm. **p < 0.01.

Given the importance of CD8^+^ T cells in colitis-related changes during anti-PD-1 immunotherapy, we evaluated the quantity of CD8^+^ T cells by immunofluorescence staining, the results showed that CD8^+^ T cells increased significantly after anti-PD-1 treatment, on the contrary, we found that CD8^+^ T cells decreased in sirolimus treatment alone and when combined with anti-PD-1 (**Figures 5A, B** **)**. We have proved that, during anti-PD-1 immunotherapy, phosphorylation of PI3K-AKT pathway, especially phosphor-AKT and phosphor-S6 were upregulated, when combined with sirolimus, the phosphorylation of AKT and S6 were both downregulated (**Figures 5C, D** **)**, indicating that sirolimus may be an alternative treatment targeting irAEs.

### Sirolimus Synergizes With Anti-PD-1 Immunotherapy *via* Inducing Tumor Cell ICD and Promoting T-Cell Infiltration

**Figure 6:**
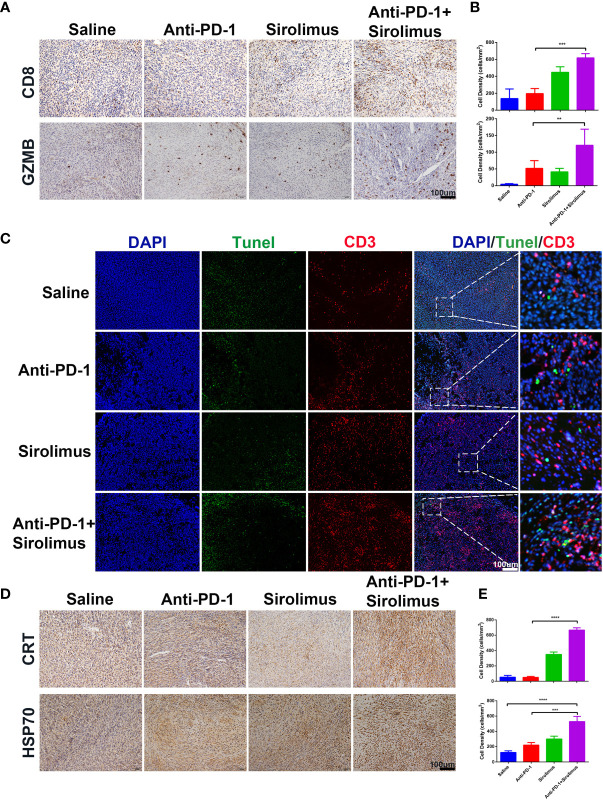
Sirolimus improved the efficacy of anti-PD-1 immunotherapy *in vivo*. **(A)** CD8 (upper) and GZMB (bottom) IHC staining of FFPE tumor sections from each group. Scale bar 200 μm. **(B)** The densities of CD8^+^ T cells (upper) and GZMB^+^ cells were quantified. The results were presented as mean ± SD (n=4). **p < 0.01; ***p < 0.001. **(C)** CD3^+^ T cells were co-visualized with TUNEL by immunofluorescence staining of FFPE tumor sections from each group. CD3 is shown in red, TUNEL in green. Scale bar 100 μm. **(D)** CRT (upper) and Hsp70 (bottom) IHC staining of FFPE tumor sections from each group. Scale bar 200 μm. **(E)** The densities of CRT^+^ (upper) and Hsp70^+^ (bottom) cells were quantified. The results were presented as mean ± SD (n=4). ***p < 0.001; ****p < 0.0001.

The antitumor effects of combination therapy involving sirolimus and anti-PD-1 were evaluated using the SMM103 subcutaneous tumor model (**Figure 4D**). The combination therapy significantly suppressed the growth of SMM103 tumors compared with saline and anti-PD-1 monotherapy. Many studies have reported the correlation between tumor infiltrating lymphocytes (TILs) and favorable prognosis in different tumor types, people who respond to single-agent PD-1 blockade therapy have a higher density of baseline CD8^+^ T cells infiltration, we investigated whether single-agent sirolimus had changed the tumor microenvironment by bringing T cells into SMM103 melanoma lesions which responded to anti-PD-1 therapy. Indeed, immunohistochemical staining of CD8 on SMM103 subcutaneous tumor indicated that sirolimus treatment significantly increased the number of CD8^+^ T cells compared with saline and anti-PD-1 monotherapy (**Figures 6A, B**, upper). We also performed IHC for the GZMB, which is associated with the cytotoxic subset of CD8^+^ T cells. The results showed an increased GZMB expression in tumors after sirolimus and combination treatment (**Figures 6A, B**, bottom), indicating that sirolimus not only increased TILs’ infiltration but also reinforced their cytotoxic activities. Through further analysis by the immunofluorescence of CD3^+^ T cells along with the apoptosis marker TUNEL, we found that tumor apoptosis was elevated after sirolimus treatment (**Figure 6C**), providing additional supporting evidence for treatment-related change in the tumor microenvironment which the number of GZMB-producing cytotoxic T cells was increased. We have proved sirolimus can induce ICD in SMM103 melanoma cells, to further characterize changes in the tumor microenvironment, we performed IHC staining of tumor biopsies of each group, we observed clear increases in cells expressing CRT and cells expressing HSP70 (**Figures 6D, E** **)**. These results showed that, through inducing ICD and recruitment of cytotoxic CD8^+^ T cells in the tumor microenvironment, sirolimus can augment anti-tumor responses of anti-PD-1.

## Discussion

In the past decades, pre-clinical experiments and clinical trials showed significant efficacy of immune checkpoint blockade in various cancers (19–21), indeed, several immune checkpoint inhibitors including anti-PD-1 have now been approved by the FDA for the treatment of melanoma, non-small-cell lung carcinoma, kidney carcinoma, gastric cancer, etc. The programmed cell death pathway is one of the most characterized immune evasion mechanisms (22). To evade the anti-tumor immune response of effector T cells, tumor cells frequently upregulate the programmed cell death pathway (23). Anti-PD-1 therapy blocks this pathway to restore the lost antitumor immunity (24–26). However, ICIs including anti-PD-1 are often systemic and may cause unwanted off-target immunological and inflammatory events known as irAEs. irAEs are not always unfavorable during treatment of ICIs, a number of studies have reported a positive association between the incidence of irAEs and clinical outcome and benefit for patients treated with ICIs (27–29). However, high-grade irAEs are often associated with severe declines in organ function and quality of life, and fatal outcomes have been reported (16, 30–32). Multiple organs and systems may affect by these adverse events (33). Skin toxicity, such as rash and vitiligo may be the most prevalent and the earliest to occur, 30%-50% of patients treated with ICIs were reported to have skin toxicities (29, 34). Gastrointestinal tract toxicities were also reported to be the most common complications during ICIs treatment (35). Cardiovascular adverse events were rare, but they were potentially the most life-threatening irAEs following ICIs treatment. Except for neurologic, hematologic and cardiac toxicities, ICIs immunotherapy should be continued with close monitoring for grade 1 toxicities. As for moderate to severe irAEs, discontinuation of ICIs treatment and initiation of high-dose corticosteroids were recommended, which definitely impair the anti-tumor responses (10, 11). Tumor progression or irAEs may cause death in some patients. In order to increase patient safety, it is currently critical to obtain a better understanding of the mechanisms underlying irAEs. The first priority is to establish pre-clinical mouse models to fully study irAEs, which will allow us to investigate how to uncouple anti-tumor immunity from hazardous irAEs.

Corticosteroids have long been used to treat autoimmune diseases due to their immunosuppressive effects. Corticosteroids have been shown to suppress the activation of effector T cells (36, 37). High-dose systemic corticosteroids were also widely used for the treatment of irAEs in patients receiving immune checkpoint inhibitors, despite the fact that they may impair anti-tumor immunity (38), long-term use of glucocorticoids can affect multiple systems, including an increased risk of infection or heart attack (39). Infliximab, one of the TNF (tumor necrosis factor) antagonists, was also used to reduce irAEs, however, TNF antagonists may cause heart failure or exacerbate existing disease (40). Sirolimus is a specific mTOR inhibitor, mTOR is a serine/threonine kinase that plays key regulatory roles in several biological processes, including cell differentiation, autophagy and proliferation and survival of normal and malignant cells, treatment of cells with sirolimus promoted G1 arrest (41–44). mTOR pathway is also a critical regulator of T cells (45), sirolimus was frequently used to maintain immune tolerance and prevent allograft rejection (46). Ultimately, sirolimus was regarded as an immunosuppressive agent. However, sirolimus was also used strategically in experimental models to enhance vaccine efficacy against viruses and tumors (47–50). It was also reported that the combination of sirolimus to anti-PD-1 could change the immune landscape in favor of allograft preservation without compromising anti-tumor efficacy (51). We hypothesized that due to mTOR pathways acting as sensors and transducers of environmental stimuli, T cells in different microenvironments may respond diversely to mTOR inhibition (52–54).

In our study, we have proved that the number of CD8^+^ T cells is increased in inflammatory colon tissues in both patients and our pre-clinical mouse model. This phenomenon is also commonly observed in other tissues affected by irAEs (16, 55). It’s crucial to investigate the origin of these CD8^+^ T cells. According to existing published researches, these CD8^+^ T cells may already be present in the healthy tissues (15), which may be the reason that skin and colon tissues are more likely to be affected by irAEs for the immense tissue-resident immune cells. Another theory is that antigens in healthy tissues are similar or even identical to antigens recognized by tumor infiltrating T cells in tumor microenvironments, Some of these tumor infiltrating T cells leaked from tumor and flowed into bloodstream, when these T cells arrived healthy tissues through blood circulation, they started to attack healthy tissues because of shared antigens (56). In our pre-clinical mouse model, we also found that sirolimus could reduce irAEs and increase ICD in the tumor microenvironment, which further accelerates the anti-tumor immune responses and so synergizes with anti-PD-1 to decrease tumor progression. It is important to recognize that our research has limitations. First and foremost, we must thoroughly investigate the safety of sirolimus, particularly in cancer patients whose conditions are complicated. It’s also worth noting that T cells in the tumor microenvironment are also regulated by PI3K-AKT-mTOR pathway. Our next project will focus on how immunotherapy affects the entire immune system. On the basis of peer-reviewed research and case studies. T cells are thought to be a prime factor in irAEs-affected tissues. However, it’s worth noting that the PD-1 receptor is not solely expressed on T cells, some of the myeloid cells expressed PD-1 as well (57), anti-PD-1 therapy may also exert influence on these myeloid cells, causing an influx of cytokines and inflammatory cells into healthy tissues or organs, which might incite damage. Following ICIs treatment, several patients generated new autoantibodies, indicating that B cells may also be associated with irAEs (58).

In summary, in this study, we developed an anti-PD-1-responsive melanoma mouse model that underwent colitis during anti-PD-1 treatment, combined with scRNA-seq data of patients, we analyze the cellular and molecular mechanisms of anti-PD-1 induced colitis, particularly focus on CD8^+^ lymphocytes, we also provided a therapeutic regimen that combined sirolimus with anti-PD-1, sirolimus played a pivotal role not only in abating immune toxicities induced by anti-PD-1 immunotherapy but in enhancing anti-tumor responses in the tumor environment. Our study provides a new treatment approach in the management of irAEs.

## Data Availability Statement

Publicly available datasets were analyzed in this study. This data can be found here: https://www.ncbi.nlm.nih.gov/geo/query/acc.cgi?acc=GSE144469.

## Ethics Statement

The animal study was reviewed and approved by Ethics Review Committee of Animal Experimentation of Sichuan University.

## Author Contributions

All authors contributed to performing the experiments, analyzing the data, and writing the manuscript. All authors contributed to the article and approved the submitted version.

## Funding

This work was supported by the following funding, (1) National Natural Science Foundation of China (No. 81773752 to HS, 22105137 to XL), (2) Key Program of the Science and Technology Bureau of Sichuan, China (No. 2021YFSY0007 to HS), (3) 1.3.5 projects for 766 disciplines of excellence, West China Hospital, Sichuan University (No. ZYYC20013 to HS), (4) China Postdoctoral Science Foundation (2020M683324 to XL), (5) Post-Doctor Research Project, West China Hospital, Sichuan University (No. 2020HXBH051 to XL).

## Conflict of Interest

Author GM, WZ, XZ, and ZZ is employed by Hangzhou Zhong Mei Hua Dong Pharmaceutical Co., Ltd.

The remaining authors declare that the research was conducted in the absence of any commercial or financial relationships that could be construed as a potential conflict of interest.

## Publisher’s Note

All claims expressed in this article are solely those of the authors and do not necessarily represent those of their affiliated organizations, or those of the publisher, the editors and the reviewers. Any product that may be evaluated in this article, or claim that may be made by its manufacturer, is not guaranteed or endorsed by the publisher.
